# Autoimmune diseases and immunosuppressive therapy in relation to the risk of glioma

**DOI:** 10.1002/cam4.2767

**Published:** 2019-12-10

**Authors:** Tareq M. Anssar, Michael F. Leitzmann, Ralf A. Linker, Christoph Meier, Claudia Becker, Susan Jick, Katharina Sahm, Michael Platten, Peter Hau, Corinna Seliger

**Affiliations:** ^1^ Wilhelm Sander‐NeuroOncology Unit and Department of Neurology Regensburg University Hospital Regensburg Germany; ^2^ Institute of Epidemiology and Preventive Medicine Regensburg University Hospital Regensburg Germany; ^3^ Basel Pharmacoepidemiology Unit Division of Clinical Pharmacy and Epidemiology Department of Pharmaceutical Sciences University of Basel Basel Switzerland; ^4^ Boston Collaborative Drug Surveillance Program Lexington United States; ^5^ Hospital Pharmacy University Hospital Basel Basel Switzerland; ^6^ Boston University School of Public Health Lexington United States; ^7^ Department of Neurology Mannheim Medical Center University of Heidelberg Mannheim Germany; ^8^ DKTK CCU Neuroimmunology and Brain Tumor Immunology German Cancer Research Center Heidelberg Germany; ^9^ Department of Neurology Heidelberg University Hospital Heidelberg Germany

**Keywords:** autoimmune diseases, glioma, immunosuppressive therapies

## Abstract

Effectors from the immune system can modulate the course and possibly the early development of gliomas. We, therefore, hypothesized that autoimmune diseases associated with increased immune‐surveillance may also modulate the risk of human glioma. To test this hypothesis, we used data from the well‐validated Clinical Practice Research Datalink (CPRD) GOLD from the UK to analyze the association of immune‐related disorders or use of immunosuppressive drugs and the risk of glioma. We identified 3112 incident glioma cases diagnosed between 1995 and 2017. We randomly selected up to 10 controls, matching them to glioma cases on age, sex, index date, general practice, and number of years of active history in the database prior to the index date. We performed conditional logistic regression analyses to estimate Odds Ratios (ORs) of glioma among those exposed to allergies, autoimmune diseases, and immunosuppressive drugs. Overall, we found no materially altered association between a history of any autoimmune disease (OR 0.98, 95% CI 0.86‐1.11), allergy (OR 0.97, 95% CI 0.89‐1.05), or use of immunosuppressive drugs and the risk of glioma. However, subgroup analyses among younger patients found a statistically significant increased risk of glioma in patients with a history of inflammatory bowel disease (IBD) (OR 2.59, 95% CI 1.31‐5.12). There was also an inverse association between asthma and risk of glioma in patients with longer survival (OR 0.73, 95% CI 0.58‐0.91) and between long‐term duration diabetes and risk of glioma (OR 0.71, 95% CI 0.53‐0.96).

## Graphical abstract

The immune system is increasingly recognized as a key player in glioma pathobiology. Small case‐control studies have found inverse associations between autoimmune diseases and glioma incidence. We performed a large case‐control study, which did not replicate these associations. However, our data indicate that inflammatory bowel disease (IBD) among patients ≤40 years are associated with an increased risk of glioma. Further studies are needed to explore the role of IBD and altered microbiota in glioma.

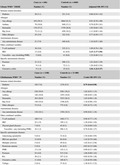

## 1 INTRODUCTION

Gliomas are primary brain tumours and glioblastoma is the most common type of glioma.^1^ Despite standard therapy with resection, combined radio‐chemotherapy, and adjuvant chemotherapy with tumor‐treating fields, glioblastomas still have a median survival of only 20.9 months.^2^ Ionizing radiation is the only known environmental factor associated with increased risk of brain tumors.^3^

Current understanding indicates various interaction mechanisms between the immune system and the central nervous system (CNS). Using specific lymphatic vessels, antigens and immune cells from the cerebral fluid drain into the deep cervical lymph nodes, thus interacting with the immune system.^4^ Furthermore, in diseases such as autoimmune encephalitis, neurodegenerative diseases, and brain tumors, immune cells from the blood migrate to the CNS.^5^, ^6^, ^7^ Antigen‐presenting cells are predominantly located in perivascular spaces, allowing T‐cell reactivation^8^ which subsequently triggers immunological events.

Despite good response rates to immunotherapy in a couple of solid tumor entities such as lung cancer or melanoma,^9^, ^10^ introduction of these therapeutics in neuro‐oncology has not yet improved survival in glioblastoma.^11^ Therefore, further understanding of tumor biology and mechanisms of immune modulation in glioblastoma is crucial for the development of new immunotherapeutic approaches.

Autoimmune diseases (AD) are typically characterized by the presence of autoreactive immune cells and the production of autoantibodies.^12^ However, T‐cells exert distinct effects in glioma and autoimmune disease. In contrast to multiple sclerosis, regulatory T‐cell (Treg) function is preserved in glioma and thus, Tregs are able to maintain peripheral tolerance in patients with glioma.^13^ This raises the question of whether the activated immune system in autoimmune disorders may be able to induce an immune‐response against transformed glioma cells that show “foreign” epitopes on their surface,^14^ which could translate into a preventive effect against glioma development and a decreased incidence of gliomas in patients with history of AD.

A number of studies have investigated the risk of brain tumors in relation to immune‐related conditions. Several studies observed a reduced risk of glioma in patients with history of allergies^15^, ^16^, ^17^, ^18^, ^19^, ^20^, ^21^ and autoimmune diseases,^15^, ^22^ while others found no significant associations.^16^, ^23^, ^24^, ^25^

In order to provide more data on these questions, we conducted a comprehensive examination of immune‐related disorders and intake of immunosuppressive drugs with regard to glioma risk, using primary‐care data from the UK

## 2 MATERIALS AND METHODS

### 2.1 Data source

The Clinical Practice Research Datalink (CPRD) GOLD is a large longitudinal database, which encompasses patient information on over 11 million patients from around 670 general practices representative of the UK population with respect to sex, age, and ethnicity. The CPRD GOLD includes medical records on over 11 million patients with acceptable quality for research based on data quality checks.^26^ The validity of information in the CPRD GOLD has been thoroughly documented and found to be of high quality for research purposes.^26^, ^27^

We received approval from the Independent Scientific Advisory Committee for Medicines and Healthcare products Regulatory Agency database research (ISAC, protocol number 16_158R). The study protocol was made available for reviewers/editors.

Data for this study were derived from CPRD primary care data obtained under license from the UK Medicines and Healthcare products Regulatory Agency. The data are provided by patients and collected by the NHS as part of their care and support. The interpretation and conclusions contained in this study are those of the authors alone.

### 2.2 Study population

#### 2.2.1 Cases

As described previously^28^, ^29^, ^30^ we used medical READ codes to identify cases. We defined cases as patients under 90 years of age, with newly diagnosed, incident glioma between 1995 and 2017. We defined the date of the first diagnosis of glioma minus 1 year as “index date”. We implemented this 1 year shift backwards in time in order to account for potential treatment of early symptoms during glioma development and before diagnosis.

We applied the same other inclusion and exclusion criteria as in our previous glioma studies, which are described in detail there.^28^, ^29^, ^30^

#### 2.2.2 Controls

The selection of controls is analogous to our previous glioma studies. We randomly selected up to 10 controls without a history of glioma in the CPRD and matched them to glioma cases on age, sex, index date, general practice, and number of years of active history in the CPRD prior to the index date.

We applied the same exclusion criteria to controls as to cases and additionally excluded controls with a recent (1 year before index date) craniotomy as some of these patients might have an unrecorded glioma diagnosis.

#### 2.2.3 Exposures

The exposures of interest in this study were AD and other immune‐related disorders identified from CPRD electronic records: The autoimmune diseases included were inflammatory bowel diseases (not specified, Crohn's disease, ulcerative colitis), Addison's disease, allergic enterocolitis, ankylosing spondylitis, antiphospholipid syndrome, autoimmune hemolytic purpura, different forms of vasculitis (including allergic purpura, Behcet's disease, Goodpasture syndrome, Churg Strauss disease, cryoglobulinaemic vasculitis, Takayasu arteritis, polyarteritis nodosa, microscopic polyangiitis, giant cell arteritis, granulomatosis with polyangiitis, other vasculitis), autoimmune connective tissue diseases (lupus erythematodes, scleroderma, Sjogren's syndrome), thyroid gland diseases (autoimmune parathyroiditis, chronic thyroiditis, Grave's disease, Hashimoto's disease, other thyroiditis), biliary cirrhosis, chronic gastritis, Guillain Barré Bannwarth syndrome, coeliac disease, Jaccoud arthropathy, juvenile pemphigoid, myasthenia gravis, microscopic colitis, pemphigus, pernicious anemia, sarcoidosis, polymyalgia rheumatica, multiple sclerosis, psoriasis, rheumatic fever, Reiter's disease, thrombocytopenic purpura, type I diabetes, vitiligo, and rheumatoid arthritis (RA). Allergies in general were included as a single variable in our analysis. We also investigated associations between asthma, dermatitis, hay fever or other specific allergies and the risk of glioma separately.

We explored autoimmune diseases as a single variable, and separately for each specific disease entity. We also evaluated combined variables for inflammatory bowel diseases, thyroid gland diseases, vasculitis, and autoimmune connective tissue diseases due to limitations of small numbers.

We further defined a single variable for T‐cell mediated diseases, which included Addison's disease, Crohn's disease, multiple sclerosis, RA, coeliac disease, Hashimoto's disease, psoriasis, type I diabetes, and sarcoidosis. We considered a patient exposed to one of the above diseases if she or he had at least one Read code for that disease recorded before the index date. Using the first listed Read code for each disease we calculated disease duration as the interval between the first record of each AD and the index date, and we categorized duration as short, intermediate or long (<5, 5‐10, >10 years).

We also assessed exposure to immunosuppressive drugs that are frequently used to treat autoimmune diseases (systemic corticosteroids, inhaled corticosteroids, topical corticosteroids, mesalazine/5‐ASA, azathioprine, mercaptopurine, methotrexate, anti‐TNF therapy, calcineurin inhibitors, interleukin inhibitors, other immunosuppressants). We defined patients who received no prescription for the drugs in question as nonusers (reference). We categorized exposure to immunosuppressive drugs based on the number of prescriptions of corticosteroids (0, 1‐4, 5‐9, ≥10) or other immunosuppressive drugs (0, 1‐19, ≥20) before the index date.

### 2.3 Statistical analyses

We conducted conditional logistic regression analyses to calculate odds ratios (ORs) with 95% confidence intervals (CIs) of glioma for exposure to autoimmune diseases, by duration of the disease and for the use of immunosuppressive drugs. We used SAS version 9.4 (SAS Institute Inc) to conduct all analyses.

We evaluated the following covariates as potential confounders: smoking status (unknown, current, past, never), body mass index (unknown, <18.5, 18.5‐24.9, 25.0‐29.9, >30.0 kg/m^2^), use of NSAIDs (none, 1‐9 prescriptions, ≥10 prescriptions), statins (none, 1‐9 prescriptions, ≥10 prescriptions), and estrogens (none, 1‐9 prescriptions, ≥10 prescriptions). We only included variables that altered the risk of glioma by >10% in the final multivariate analyses. We performed subgroup analyses after stratification by glioma subtype, age, and sex.

We also tested linear trends of autoimmune disease duration and prescriptions of immunosuppressive drugs using a Wald test analogous to our previous descriptions.^29^, ^30^ When we investigated specific autoimmune diseases, we corrected for multiple testing controlling the False Discovery Rate at 5% according to the Benjamini‐Hochberg procedure.^31^

## 3 RESULTS

### 3.1 Basic characteristics of cases and controls

We identified 3112 patients with incident glioma and 31 120 matched controls who met the inclusion criteria. Mean (SD) length of history in the database before the index date was 11.6 years (±5.6 years). On average, there were 9.1 practice visits per year in cases and 8.8 in controls. See Table 1 for information on basic characteristics of cases and controls are. We do not display cell sizes less than 5 in compliance with CPRD guidelines.

**Table 1:** Demographic characteristics in cases and controls

	Cases (n = 3112)	Controls (n = 31 120)	Crude OR (95% CI)
	Number (%)	Number (%)	
Sex			
Male	1713 (55.0)	17 130 (55.0)	
Female	1399 (45.0)	13 990 (45.0)	
Age class			
0‐39	620 (19.9)	6194 (19.9)	
40‐59	1011 (32.5)	10 093 (32.4)	
>=60	1481 (47.6)	14 833 (47.7)	
Mean age (y)			
Mean (SD)	54.7 (19.4)	54.7 (19.4)	
Length of history before index date (y)			
Mean (SD)	11.6 (5.6)	11.6 (5.6)	
BMI (kg/m^2^)			
<18.5	14 (0.5)	376 (1.2)	**0.36 (0.21‐0.62)**
18.5‐24.9	907 (29.2)	8815 (28.3)	1.00 (reference)
25‐29.9	927 (29.8)	8986 (28.9)	1.01 (0.91‐1.11)
>=30	532 (17.1)	5217 (16.8)	1.00 (0.89‐1.12)
Unknown	732 (23.5)	7726 (24.8)	**0.88 (0.78‐1.00)**
Smoking status			
Smoker	472 (15.2)	5172 (16.6)	**0.84 (0.75‐0.94)**
Past smoker	738 (23.7)	7380 (23.7)	0.94 (0.85‐1.03)
Non‐smoker	1482 (47.6)	13 781 (44.3)	1.00 (reference)
Unknown	420 (13.5)	4787 (15.4)	**0.71 (0.61‐0.83)**
Glioma subtype			
Lower grade glioma WHO ° I/II/III	646 (20.7)	6460 (20.8)	
Glioblastoma WHO ° IV	1348 (43.3)	13 480 (43.3)	
Not specified	1118 (35.9)	11 180 (35.9)	
Comorbidities			
CHF	29 (0.9)	508 (1.6)	**0.56 (0.38‐0.81)**
MI	82 (2.6)	1048 (3.4)	**0.77 (0.61‐0.97)**
Stroke	105 (3.4)	1182 (3.8)	0.88 (0.72‐1.08)
IHD	225 (7.2)	2392 (7.7)	0.93 (0.80‐1.08)
Hyperlipidemia	305 (9.8)	3306 (10.6)	0.90 (0.79‐1.03)
DVT	58 (1.9)	460 (1.5)	1.27 (0.96‐1.67)
Gout	101 (3.3)	1133 (3.6)	0.88 (0.72‐1.09)
Renal disease	85 (2.7)	778 (2.5)	1.10 (0.87‐1.38)
COPD	73 (2.4)	881 (2.8)	0.82 (0.64‐1.05)
Number of practice visits			
0‐9	2159 (69.4)	22 473 (72.2)	
20‐29	204 (6.6)	1744 (5.6)	
>=30	85 (2.7)	865 (2.8)	
Mean (SD)	9.09 (9.0)	8.76 (9.0)	
Comedication			
Number of prescriptions			
Statins			
0	2557 (82.2)	25 735 (91.0)	1.00 (reference)
1‐9	140 (4.5)	1251 (4.0)	1.14 (0.94‐1.36)
>=10	415 (13.3)	4134 (13.3)	1.02 (0.90‐1.16)
NSAIDs			
0	1313 (42.2)	13 616 (43.8)	1.00 (reference)
1‐9	1400 (45.0)	13 496 (43.4)	1.09 (1.00‐1.18)
>=10	399 (12.8)	4008 (12.9)	1.046 (0.92‐1.19)
Estrogens (women only)			
0	1076 (76.9)	10 837 (77.5)	1.00 (reference)
1‐9	233 (16.7)	2242 (16.0)	1.05 (0.90‐1.24)
>=10	90 (6.4)	911 (6.4)	1.00 (0.79‐1.27)

Note

Bold indicates significant value (*P* < .05).

Matching variables: calendar time (same index date), age (same year of birth), sex, general practice, and number of years of active history in the database prior to the index date.

Abbreviations: BMI, body mass index; CHF, congestive heart failure; CI, confidence interval; COPD, chronic obstructive pulmonary disease; DVT, deep vein thrombosis; IHD, ischemic heart disease; MI, myocardial infarction; NSAIDs, nonsteroidal anti‐inflammatory drugs; OR, odds ratio; SD, standard deviation.

There were more male than female cases (55.0% male and 45.0% female), and the mean age was 54.7 years. There were 646 cases (20.8%) with lower grade glioma (WHO grade I/II/III), 1348 cases (43.3%) with glioblastoma (WHO grade IV), and 1118 cases (35.9%) with glioma that was not further specified.

In univariate analyses, low BMI (underweight) was inversely associated (OR 0.36; 95% CI 0.21‐0.62) to the risk of glioma compared to normal weight. Being a current smoker (OR 0.84, 95% CI 0.75‐0.94) was also associated with a reduced risk of glioma compared to nonsmokers. Heart failure (OR 0.56, 95% CI 0.38‐0.81) and past myocardial infarction (OR 0.77, 95% CI 0.61‐0.97) were inversely associated to the incidence of glioma, whereas a history of stroke, chronic obstructive pulmonary disease, hyperlipidemia, deep vein thrombosis, coronary heart disease, gout, and renal disease were not associated with an altered OR. Comedication with statins, NSAIDs, or estrogens (women only) also showed no significant relation to glioma risk. When we stratified by sex, congestive heart failure was associated with a reduced risk in male patients (OR 0.54, 95% CI 0.33‐0.88). In female patients with glioma, deep vein thrombosis was observed more often than in controls (OR 1.65, 95% CI 1.14‐2.37). See Table 1 for details.

### 3.2 Multivariate models

#### 3.2.1 Autoimmune diseases

We observed 282 (9.06%) cases with a recorded history of any autoimmune disease. There was no association between patients with any “autoimmune disease” and glioma risk (OR 0.98, 95% CI 0.86‐1.11). We also investigated combined variables for any inflammatory bowel disease (OR 1.28, 95% CI 0.95‐1.71), any autoimmune connective tissue disease (OR 1.10, 95% CI 0.50‐2.41), any thyroid gland disease (OR 1.15, 95% CI 0.59‐2.21), and any vasculitis (OR 1.07, 95% CI 0.79‐1.47), none of which were associated with risk of glioma.

When we analyzed the data for the various autoimmune diseases separately (Table 2), none of them were related to an altered risk of glioma. There were also no statistically significant associations between Crohn's disease (OR 0.96, 95% CI 0.48‐1.90), ulcerative colitis (OR 1.27, 95% CI 0.85‐1.88), or inflammatory bowel disease not further specified (OR 1.27, 95% CI 0.81‐1.98), and the risk of glioma. A statistically nonsignificant reduced risk of glioma of >20% was observed for type I diabetes, rheumatic fever, sarcoidosis, and coeliac disease. We observed a statistically nonsignificant increased risk for pernicious anemia, Hashimoto, Sjogren's syndrome, and vitiligo (Table 2).

**Table 2:** Risk of glioma in patients with immune‐related disorders and autoimmune diseases

	Cases (n = 3112)	Controls (n = 31 120)	Adjusted OR (95% CI)
	Number (%)	Number (%)	
Immune‐related disorders			
Diabetes	204 (5.6)	2295 (7.4)	0.86 (0.73‐1.00)
Allergies			
Any allergy^a^	1219 (39.2)	12 291 (39.5)	0.97 (0.89‐1.05)
Asthma	342 (11.0)	3621 (11.6)	0.91 (0.81‐1.03)
Dermatitis	828 (26.6)	8093 (26.0)	1.02 (0.94‐1.11)
Hay fever	299 (9.6)	2843 (9.1)	1.04 (0.91‐1.18)
Other allergies	157 (5.0)	1660 (5.3)	0.93 (0.78‐1.10)
Autoimmune diseases			
Any autoimmune disease	282 (9.1)	2871 (9.2)	0.98 (0.86‐1.11)
Other combined variables			
T‐cell mediated	190 (6.1)	2113 (6.8)	0.89 (0.76‐1.04)
Connective tissue disease	7 (0.2)	63 (0.2)	1.10 (0.50‐2.41)
IBD	52 (1.7)	406 (1.3)	1.28 (0.95‐1.71)
Thyroid gland diseases	10 (0.3)	87 (0.3)	1.15 (0.59‐2.21)
Vasculitis—any (including PMR)	46 (1.5)	423 (1.4)	1.07 (0.79‐1.47)
Vasculitis (without PMR)	11 (0.4)	135 (0.4)	0.81 (0.44‐1.49)
Specific autoimmune diseases			
Ankylosing spondylitis	8 (0.3)	71 (0.2)	1.12 (0.54‐2.33)
Coeliac disease	6 (0.2)	82 (0.3)	0.73 (0.32‐1.67)
Crohn's disease	9 (0.3)	93 (0.3)	0.96 (0.48‐1.90)
Diabetes type I	5 (0.2)	82 (0.3)	0.59 (0.24‐1.46)
Hashimoto	5 (0.2)	22 (0.1)	2.30 (0.87‐6.09)
IBD‐not specified	22 (0.7)	175 (0.6)	1.27 (0.81‐1.98)
Multiple sclerosis	9 (0.3)	85 (0.3)	1.06 (0.53‐2.11)
Pernicious anemia	15 (0.5)	104 (0.3)	1.46 (0.85‐2.52)
PMR	35 (1.1)	288 (0.9)	1.20 (0.84‐1.72)
Psoriasis	104 (3.3)	1136 (3.7)	0.92 (0.75‐1.13)
Rheumatic fever	5 (0.2)	75 (0.2)	0.66 (0.27‐1.63)
RA	53 (1.7)	596 (1.9)	0.89 (0.67‐1.18)
Sarcoidosis	6 (0.2)	81 (0.3)	0.72 (0.31‐1.65)
Sjogren's syndrome	5 (0.2)	31 (0.1)	1.54 (0.60‐3.99)
Ulcerative colitis	28 (0.9)	218 (0.7)	1.27 (0.85‐1.88)
Vitiligo	11 (0.4)	82 (0.3)	1.31 (0.70‐2.46)

Note

Adjusted for: smoking and BMI.

Matching variables: calendar time (same index date), age (same year of birth), sex, general practice, and number of years of active history in the database prior to the index date.

Abbreviations: CI, confidence interval; IBD, inflammatory bowel disease; OR, odds ratio; PMR, polymyalgia rheumatica; RA, rheumatoid arthritis.

a Any allergy: combined variable for asthma, dermatitis, hay fever and other allergies.

Upon stratification by sex, we observed no divergent results between males and females (data not shown).

#### 3.2.2 Allergies

Of all patients with glioma, 1219 (39.17%) had a history of some allergic condition (Table 2). We observed no altered glioma risk among patients with “any allergy” (OR 0.97, 95% CI 0.89‐1.05) or with any of the specific allergic conditions.

#### 3.2.3 Glioma subtypes

We also analyzed the different glioma subtypes separately to see if the results differed according to type (Table 3). For methodologic reasons (Read Codes do not always differentiate well between WHO grade I, II, and III glioma), we distinguished between grades I/II/III (lower grade) and grade IV glioma.

**Table 3:** Risk of glioma subtypes in patients with immune‐related disorders and autoimmune diseases

Glioma WHO ° I/II/III	Cases (n = 646)	Controls (n = 6460)	Adjusted OR (95% CI)
	Number (%)	Number (%)	
Immune‐related disorders			
Diabetes	20 (3.1)	221 (3.4)	0.88 (0.54‐1.42)
Allergies			
Any allergy	254 (39.3)	2664 (41.2)	0.91 (0.76‐1.08)
Asthma	70 (10.8)	848 (13.1)	0.78 (0.59‐1.01)
Dermatitis	176 (27.2)	1729 (26.8)	1.02 (0.84‐1.24)
Hay fever	72 (11.2)	658 (10.2)	1.11 (0.85‐1.44)
Other allergies	25 (3.9)	322 (5.0)	0.77 (0.50‐1.17)
Autoimmune diseases			
Any autoimmune disease	51 (7.9)	440 (6.8)	1.18 (0.87‐1.60)
Other combined variables			
T‐cell mediated	36 (5.6)	332 (5.1)	1.09 (0.76‐1.56)
IBD	13 (2.0)	61 (0.9)	**2.15 (1.17‐3.96)**
Vasculitis—any (including PMR)	5 (0.8)	41 (0.6)	1.22 (0.48‐3.11)
Specific autoimmune diseases			
Psoriasis	21 (3.3)	208 (3.2)	1.01 (0.63‐1.59)
RA	9 (1.4)	72 (1.1)	1.26 (0.62‐2.54)
Ulcerative colitis	9 (1.4)	35 (0.5)	**2.56 (1.22‐5.37)**

Glioblastoma WHO ° IV	Cases (n = 1348)	Controls (n = 13 480)	Crude OR (95% CI)
	Number (%)	Number (%)	
Immune‐related disorders			
Diabetes	105 (7.8)	1276 (9.5)	**0.79 (0.64‐0.98)**
Allergies			
Any allergy	538 (39.9)	5281 (39.2)	1.02 (0.91‐1.15)
Asthma	146 (10.8)	1455 (10.8)	1.00 (0.83‐1.20)
Dermatitis	363 (26.9)	3533 (26.2)	1.03 (0.90‐1.18)
Hay fever	140 (10.4)	1196 (8.9)	1.18 (0.98‐1.43)
Other allergies	73 (5.4)	775 (5.8)	0.92 (0.72‐1.19)
Autoimmune diseases			
Any autoimmune disease	136 (10.1)	1394 (10.3)	0.98 (0.81‐1.18)
Other combined variables			
T‐cell mediated	100 (7.4)	1042 (7.7)	0.96 (0.77‐1.19)
IBD	25 (1.9)	199 (1.5)	1.28 (0.84‐1.94)
Thyroid gland diseases	7 (0.5)	45 (0.3)	1.54 (0.69‐3.42)
Vasculitis—any (including PMR)	16 (1.2)	206 (1.5)	0.76 (0.46‐1.27)
Specific autoimmune diseases			
Ankylosing spondylitis	5 (0.4)	33 (0.2)	1.54 (0.60‐3.95)
IBD‐not specified	12 (0.9)	84 (0.6)	1.45 (0.79‐2.66)
Multiple sclerosis	5 (0.4)	49 (0.4)	1.02 (0.41‐2.56)
Pernicious anemia	5 (0.4)	61 (0.5)	0.82 (0.33‐2.06)
PMR	13 (1.0)	142 (1.1)	0.90 (0.51‐1.60)
Psoriasis	54 (4.0)	515 (3.8)	1.06 (0.80‐1.42)
RA	28 (2.1)	327 (2.4)	0.85 (0.58‐1.26)
Ulcerative colitis	11 (0.8)	100 (0.7)	1.10 (0.59‐2.06)
Vitiligo	5 (0.4)	37 (0.3)	1.30 (0.51‐3.32)

Note

Bold indicates significant value (*P* < .05).

Adjusted for: smoking and BMI.

Matching variables: calendar time (same index date), age (same year of birth), sex, general practice, and number of years of active history in the database prior to the index date.

Abbreviations: CI, confidence interval; IBD, inflammatory bowel disease; OR, odds ratio; PMR, polymyalgia rheumatica; RA, rheumatoid arthritis.

In WHO grades I/II/III gliomas, IBD and specifically ulcerative colitis, were the only exposures that were associated with statistically significantly increased ORs for glioma (OR 2.15, 95% CI 1.17‐3.96 for IBD, and OR 2.56, 95% CI 1.22‐5.37 for ulcerative colitis). When we took multiple testing into account, the results were no longer statistically significant.

With regard to the risk of glioblastoma, we did not observe relations to autoimmune diseases in general (OR 0.98, 95% CI 0.81‐1.18), nor did we observe associations between any of the other specific autoimmune diseases and risk of glioblastoma, except for diabetes (combined variable for type I and type II) (OR 0.79, 95% CI 0.64‐0.98). The result was not statistically significant after correction for multiple comparisons.

### 3.3 Restriction to young patients and patients with longer survival

The two significant associations observed in this study were found in WHO grade I/II/III glioma cases with a history of IBD and ulcerative colitis, both of which tend to occur in younger patients. Therefore, we conducted further analysis restricted to cases and their corresponding controls who were below 40 years of age. Among those, patients with a record of IBD had an increased risk of glioma (OR 2.59, 95% CI 1.31‐5.12, compared to 1.28 among all glioma patients). See Table 3 for details.

When we restricted the analysis of all gliomas to patients with an overall survival of ≥20 months, again inflammatory bowel disease was associated with an elevated risk of glioma after correction for multiple testing (OR 2.10, 95% CI 1.30‐3.38). There was also an increased risk associated with coeliac disease (OR 3.72, 95% CI 1.32‐10.46). Furthermore, in this subgroup, asthma was inversely related to the risk of glioma (OR 0.73, 95% CI 0.58‐0.91). After correction for multiple testing, both effects remained statistically significant.

### 3.4 Disease duration

To assess the influence of AD duration on the risk of glioma, we looked at different times from autoimmune disease onset to index date (Table 4). We observed no effect of disease duration (<5, 5‐10, ≥10 years) on glioma for autoimmune diseases, allergies, or specific allergic conditions. However, recent onset (<5 years) of inflammatory bowel disease (OR 1.69, 95% CI 1.04‐2.76) or ulcerative colitis (OR 2.24, 95% CI 1.08‐4.63) was associated with an increased risk of glioma. We observed a trend for a reduced risk of glioma in patients with longer duration of diabetes.

**Table 4:** Risk of glioma in patients with immune‐related disorders and autoimmune diseases by time since diagnosis

	Cases (n = 3112)	Controls (n = 31 120)	Adjusted OR (95% CI)
	Number (%)	Number (%)	
*Immune‐related disorders*			
Diabetes			
0	2908 (93.4)	28828 (92.6)	1
<5 y	100 (3.2)	942 (3.0)	1.03 (0.83‐1.28)
5‐10 y	54 (1.7)	678 (2.2)	0.76 (0.58‐1.02)
>10 y	50 (1.6)	672 (2.2)	**0.71 (0.53‐0.96)**
*Allergies*			
Any allergy			
0	1904 (61.2)	18 910 (60.8)	1
<5 y	321 (10.3)	3078 (9.9)	1.02 (0.90‐1.16)
5‐10 y	321 (10.3)	3175 (10.2)	0.98 (0.86‐1.12)
>10 y	566 (18.2)	5957 (19.1)	0.91 (0.82‐1.08)
Asthma			
0	2776 (89.2)	27 544 (88.5)	1
<5 y	63 (2.0)	708 (2.3)	0.86 (0.66‐1.12)
5‐10 y	77 (2.5)	842 (2.7)	0.88 (0.69‐1.12)
>10 y	196 (6.3)	2026 (6.5)	0.94 (0.80‐1.10)
Dermatitis			
0	2285 (73.4)	23 047 (74.1)	1
<5 y	272 (8.7)	2491 (8.0)	1.09 (0.95‐1.25)
5‐10 y	235 (7.6)	2275 (7.3)	1.03 (0.89‐1.19)
>10 y	320 (10.3)	3307 (10.6)	0.96 (0.84‐1.10)
Hay fever			
0	2816 (90.5)	28 300 (90.9)	1
<5 y	82 (2.6)	747 (2.4)	1.08 (0.86‐1.36)
5‐10 y	85 (2.7)	715 (2.3)	1.18 (0.94‐1.49)
>10 y	129 (4.2)	1358 (4.4)	0.93 (0.77‐1.13)
Other allergies			
0	2959 (95.1)	29 497 (94.8)	1
<5 y	48 (1.5)	516 (1.7)	0.91 (0.68‐1.23)
5‐10 y	42 (1.4)	460 (1.5)	0.89 (0.64‐1.23)
>10 y	63 (2.0)	647 (2.1)	0.95 (0.73‐1.25)
*Autoimmune diseases*			
Any autoimmune disease			
0	2831 (91.0)	28 263 (90.8)	1
<5 y	71 (2.3)	802 (2.6)	0.88 (0.69‐1.13)
5‐10 y	76 (2.4)	648 (2.1)	1.17 (0.92‐1.49)
>10 y	134 (4.3)	1407 (4.5)	0.95 (0.79‐1.14)
*Other combined variables*			
IBD			
0	3060 (98.3)	30 716 (98.7)	1
<5 y	19 (0.6)	113 (0.4)	1.69 (1.04‐2.76)
5‐10 y	10 (0.3)	83 (0.3)	1.21 (0.63‐2.33)
>10 y	23 (0.7)	208 (0.7)	1.10 (0.71‐1.69)
Vasculitis—any (including PMR)			
0	3066 (98.5)	30 698 (98.6)	1
<5 y	18 (0.6)	189 (0.6)	0.94 (0.57‐1.53)
5‐10 y	14 (0.5)	130 (0.4)	1.07 (0.61‐1.86)
>10 y	14 (0.5)	103 (0.3)	1.34 (0.77‐2.36)
*Specific autoimmune diseases*			
IBD‐not specified			
0	3090 (99.3)	30 946 (99.4)	1
<5 y	9 (0.3)	65 (0.2)	1.40 (0.69‐2.82)
5‐10 y	6 (0.2)	42 (0.1)	1.43 (0.61‐3.36)
>10 y	7 (0.2)	67 (0.2)	1.06 (0.48‐2.30)
PMR			
0	3077 (98.9)	30 832 (99.1)	1
<5 y	16 (0.5)	144 (0.5)	1.10 (0.65‐1.85)
5‐10 y	11 (0.4)	85 (0.3)	1.28 (0.68‐2.41)
>10 y	8 (0.3)	59 (0.2)	1.35 (0.64‐2.84)
Psoriasis			
0	3009 (96.7)	29 991 (96.4)	1
<5 y	25 (0.8)	299 (1.0)	0.84 (0.56‐1.27)
5‐10 y	25 (0.8)	256 (0.8)	0.98 (0.64‐1.47)
>10 y	53 (1.7)	574 (1.8)	0.92 (0.69‐1.23)
RA			
0	3059 (98.3)	30 527 (98.1)	1
<5 y	13 (0.4)	163 (0.5)	0.79 (0.45‐1.39)
5‐10 y	16 (0.5)	145 (0.5)	1.09 (0.65‐1.83)
>10 y	24 (0.8)	285 (0.9)	0.85 (0.56‐1.29)
Ulcerative colitis			
0	3084 (99.1)	30 902 (99.3)	1
<5 y	9 (0.3)	41 (0.1)	2.24 (1.08‐4.63)
5‐10 y	5 (0.2)	52 (0.2)	0.95 (0.38‐2.39)
>10 y	14 (0.5)	125 (0.4)	1.09 (0.63‐1.90)

Note

Bold indicates significant value (*P* < .05).

Adjusted for: smoking and BMI.

Matching variables: calendar time (same index date), age (same year of birth), sex, general practice, and number of years of active history in the database prior to the index date.

Abbreviations: CI, confidence interval; IBD, inflammatory bowel disease; OR, odds ratio; PMR, polymyalgia rheumatica; RA, rheumatoid arthritis.

### 3.5 Corticosteroids and immunosuppressive drugs

There was no altered glioma risk associated with use of corticosteroids, systemic corticosteroids, or use of any immunosuppressive drug. The risk did not change for any of these exposures when we evaluated the effect according to duration of use (Table 5).

**Table 5:** Risk of glioma in relation to corticosteroids and other immunosuppressive drugs

	Cases (n = 3112)	Controls (n = 31 120)	Adjusted OR (95% CI)
	Number (%)	Number (%)	
Inhalative corticosteroids			
No prescription	2726 (87.6)	27 073 (87.0)	1
Any prescription	386 (12.4)	4047 (13.0)	0.93 (0.83‐1.04)
1‐4 prescriptions	154 (5.0)	1429 (4.6)	1.05 (0.88‐1.25)
5‐9 prescriptions	40 (1.3)	549 (1.8)	0.70 (0.51‐0.97)
>=10 prescriptions	192 (6.2)	2069 (6.7)	0.91 (0.78‐1.06)
Systemic corticosteroids			
No prescription	2312 (74.3)	23 261 (74.8)	1
Any prescription	800 (25.7)	7859 (25.3)	1.02 (0.93‐1.11)
1‐4 prescriptions	617 (19.8)	6004 (19.3)	1.03 (0.93‐1.13)
5‐9 prescriptions	89 (2.9)	883 (2.8)	1.01 (0.81‐1.27)
>=10 prescriptions	94 (3.0)	972 (3.1)	0.97 (0.78‐1.21)
Topic corticosteroids			
No prescription	1235 (39.7)	13 058 (42.0)	1
Any prescription	1877 (60.3)	18 062 (58.0)	**1.09 (1.01‐1.18)**
1‐4 prescriptions	1198 (38.5)	11 351 (36.5)	**1.11 (1.01‐1.21)**
5‐9 prescriptions	323 (10.4)	3118 (10.0)	1.08 (0.94‐1.23)
>=10 prescriptions	356 (11.4)	3593 (11.6)	1.04 (0.91‐1.18)
Other immunosuppressive drugs			
Any immunosuppressive drug			
No prescription	3042 (97.8)	30 429 (97.8)	1
Any prescription	70 (2.3)	691 (2.2)	1.01 (0.79‐1.30)
1‐19 prescriptions	40 (1.3)	435 (1.4)	0.92 (0.66‐1.27)
>=20 prescriptions	30 (1.0)	256 (0.8)	1.16 (0.80‐1.71)
Mesalazine/5‐ASA			
No prescription	3063 (98.4)	30 673 (98.6)	1
Any prescription	49 (1.6)	447 (1.4)	1.08 (0.80‐1.46)
1‐19 prescriptions	26 (0.8)	256 (0.8)	1.01 (0.67‐1.51)
>=20 prescriptions	23 (0.7)	191 (0.6)	1.18 (0.77‐1.83)
Azathioprine			
No prescription	3101 (99.7)	30 968 (99.5)	1
Any prescription	11 (0.4)	152 (0.5)	0.72 (0.39‐1.33)
1‐19 prescriptions	5 (0.2)	88 (0.3)	0.56 (0.23‐1.38)
>=20 prescriptions	6 (0.2)	64 (0.2)	0.95 (0.41‐2.20)
Methotrexate			
No prescription	3095 (99.5)	30 914 (99.3)	1
Any prescription	17 (0.6)	206 (0.7)	0.82 (0.50‐1.36)
1‐19 prescriptions	5 (0.2)	86 (0.3)	0.58 (0.24‐1.44)
>=20 prescriptions	12 (0.4)	120 (0.4)	1.00 (0.55‐1.81)
Other immunosuppressive drugs			
No prescription	3085 (99.1)	30 785 (98.9)	1
Any prescription	27 (0.9)	335 (1.1)	0.80 (0.54‐1.19)
1‐19 prescriptions	9 (0.3)	153 (0.5)	0.58 (0.30‐1.14)
>=20 prescriptions	18 (0.6)	182 (0.6)	0.99 (0.61‐1.61)

Note

Bold indicates significant value (*P* < .05).

Adjusted for: smoking and BMI.

Matching variables: calendar time (same index date), age (same year of birth), sex, general practice, and number of years of active history in the database prior to the index date.

## 4 DISCUSSION

In this population‐based case‐control study, autoimmune diseases, allergies, and use of immunosuppressive agents were not related to an altered risk of glioma. This finding is consistent with a couple of published studies that came to the same conclusion.^16^, ^25^ However, only few studies specifically addressed glioma risk in relation to subgroups of autoimmune diseases. A cohort study that included around 4.5 million US veterans found no significant association between autoimmune diseases in general or any of the other specific autoimmune conditions that were investigated, including asthma, multiple sclerosis, ulcerative colitis, Crohn's disease, and the risk of glioma.^16^ A Swedish retrospective cohort study based on the Swedish Hospital Discharge Register also found no association between any of the 33 autoimmune diseases examined, including RA, multiple sclerosis, ulcerative colitis, Crohn's diseases, and risk of glioma.^25^

However, some publications that have investigated possible environmental influences on glioma have found a lower risk in patients with a history of AD.^15^, ^22^ One of them was a case‐control study with 489 patients based on data obtained in three hospitals in the United States. The authors described a lower glioma risk in patients with autoimmune diseases in general, however, no association was found with specific autoimmune disease including RA, multiple sclerosis, lupus erythematosus, and pernicious aaemia.^15^

Interestingly, in our study there was a suggestion of an elevated risk of glioma among patients with a history of IBD, when we just examined patients with lower grade glioma (LGG) (WHO I, II, and III), patients with better overall survival, or patients below the age of 40 years. This contrasts with the results from other studies, which found no significant relation between glioma risk and the history of the inflammatory bowel disease.^16^, ^25^ However, those studies did not conduct analyses by specific subgroups of patients. Separate analyses for LGG and high‐grade gliomas (HGG) were performed in only one retrospective cohort study from Sweden.^25^ In contrast to our study, the authors of that study applied another categorization with regard to glioma grades. They defined LGG as WHO grade I and II, whereas HGG was defined as WHO grade III and IV (with only few cases in the low grade glioma subgroup).^25^ Of note, categorizing WHO glioma grades as I/II/III vs grade IV may be more appropriate on a genetic level than comparing WHO grade I/II vs grade III/IV, which may explain the null results in the Swedish study. Most WHO grade II and III gliomas are almost exclusively isocitrate dehydrogenase (IDH)‐mutated, whereas glioblastoma is not and IDH mutation and occurs more often in younger patients.^32^

Several mechanisms could explain an association between IBD and the microbiota on the brain. IBD such as Crohn's disease and ulcerative colitis are characterized by impaired intestinal barrier function.^33^ Such impaired barrier function may enable certain bacterial components to reach the intestinal epithelium, known as “bacterial translocation”, a mechanism that is hypothesized to play a role in carcinogenesis.^34^ Patients with ulcerative colitis show increased levels of serum transforming growth factor‐β1 (TGF‐ β1).^35^, ^36^ Another study found that TGF‐β production was increased in Clostridium‐colonized mice, and that formation of Tregs was induced,^37^ indicating an involvement of the intestinal microbiota in the modulation of the peripheral immune system. In glioma patients, TGF‐β has been shown to be upregulated and to take part importantly in glioma initiation and proliferation.^38^ Other mechanisms that could explain how alterations of the microbiota can affect the brain involve activation of the vagus nerve,^39^ neuroimmune pathways,^40^ microbial metabolites,^41^ and microbial‐derived neurotransmitters.^42^ For example, microbiota‐derived short‐chain fatty acids control microglia maturation and function.^41^

In solid tumors other than malignant glioma, increasing evidence suggests meaningful associations between alterations of the intestinal microbiota and colorectal carcinoma,^43^, ^44^, ^45^, ^46^ hepatocellular carcinoma,^47^, ^48^ and breast cancer,^49^ among others. Furthermore, it is well documented that the microbiota is involved in CNS‐pathologies such as Parkinson's disease^50^ or multiple sclerosis.^51^ A recent mouse model showed that increased intestinal Th17 cells and aggravation of experimentally induced autoimmune encephalomyelitis by high salt diet can be prevented by administration of certain lactobacillus species.^52^ It is therefore conceivable that alterations of the intestinal microbiota have a part in the pathogenesis of lower‐grade glioma.

Tregs play an important role in the pathophysiology of glioma by contributing to an immunosuppressive milieu.^53^ To account for alterations in T‐cell function and to include more cases of autoimmune diseases of a common pathophysiology in our analysis, we investigated the risk of glioma in patients with a history of AD, which are considered T‐cell mediated. However, no particular association was observed. A possible explanation is that even AD such as multiple sclerosis, which are considered T‐cell mediated, involve numerous other elements of the immune system.^54^ A joint examination of these diseases on the premise that they share a common pathophysiological pathway may therefore be too simplistic.

We observed no overall association between a diagnosis of allergy in general or different allergies and glioma risk in our study. However, we found an inverse relation between asthma and incidence of glioma in patients below 40 years of age after correction for multiple testing. Several other case‐control studies,^15^, ^17^, ^55^, ^56^, ^57^, ^58^, ^59^ cohort studies 16, 23, 24, and meta‐analyses 18, 20, 21 have already investigated the relation between allergic conditions and the risk of glioma. Most case‐control studies observed an inverse association between some allergic disease and the risk of glioma.^15^, ^17^, ^56^, ^57^, ^58^, ^59^

For example, in a study including 489 patients with glioma, the authors observed an inverse association between the history of any allergy, asthma, and allergy to chemicals, and the risk of glioma.^15^ Allergies and infections were related to a reduced risk of glioma in a case‐control study based on data from eight hospitals in six countries including 1178 cases.^17^

On the other hand, there is also a German case‐control study with 366 glioma cases which did not find statistically significant associations.^55^

Most of these studies differ from ours, because they were interview‐ or questionnaire‐based and might therefore have been prone to reporting bias. Additionally, information was often provided by proxy‐respondents and not by the cases themselves. It is conceivable, that underreporting of allergy/atopy occurs in cases more frequently than in controls due to cognitive impairment of patients with brain tumors. Proxy respondents might not know all allergic conditions. In our study medical records exist only if diagnoses were reported to the physician. Our data indicate that, before the index date, controls might be in poor health compared to cases as they are more likely to have a history of myocardial infarction and congestive heart failure. Underreporting of less serious issues such as allergic conditions seems plausible in sick persons, which could possibly mute inverse associations observed in previous case‐control‐studies. However, in our study, prevalence of any allergy was 39.2% in cases and 39.5% in controls which is similar to the prevalence of allergic conditions in controls in a couple of other case‐control studies.^15^, ^58^, ^59^

In general, cohort studies are more valid in providing information on causal relationships. The first cohort study that investigated the association between allergies and the risk of glioma included persons from the Swedish Twin Registry birth cohort who were grouped into cohorts I‐III according to the year of birth.^24^ In cohorts I and III they observed a reduced hazard ratio after the history of allergy.^24^ However, results were not significant and there were only six and three exposed cases in cohorts I and III respectively. Cohort II showed no association with a slightly increased hazard ratio.^24^

In a cohort of male US veterans with 192 out of 4383 glioma cases (4.38%) exposed to allergy/atopy, there was a nonsignificant correlation between allergy with latency >2 years and risk of brain tumors.^16^ A statistically significant trend of decreasing risk of brain cancer was observed with longer latency of allergy.^16^ Another cohort study based on the Swedish population which investigated the risk of various cancers including brain cancer observed no significant association with regard to the risk of brain tumors.^23^ Another cohort study in Taiwan found an increased risk of brain tumors after history of allergic rhinitis or asthma.^60^

Three meta‐analyses which included most of the case‐control studies cited above also found an inverse relation between allergy and glioma.^18^, ^20^, ^21^

Three other studies based on prospective cohorts found inverse associations of increased^61^, ^62^ or borderline^63^ pre‐diagnostic serum IgE levels and the risk of glioma and thus provide further evidence for the involvement of the immune system.

Gliomas are characterized by an immunosuppressive microenvironment and systemic immunosuppression.^53^ Therefore, it is conceivable that immunosuppression itself might be a tumor promoting factor. However, in our analysis no relation between immunosuppressive therapies and the incidence of gliomas became apparent. To date, few studies have systematically investigated the risk of glioma with regard to prescription of immunosuppressants. Our findings are in accordance with a meta‐analysis, which suggested no increased rates of brain cancer in patients with immunosuppressive therapy after organ transplantation, whereas incidence of not specified brain cancer was higher in patients with acquired immune deficiency syndrome.^64^ There is also evidence that risk of other tumor entities is associated with duration of immunosuppressive therapy.^65^ In contrast, in our study, duration of treatment with immunosuppressive drugs was not associated with the incidence of glioma. Further stratification was limited because only few cases could be identified that were exposed to individual immunosuppressive drugs.

We observed a slightly reduced risk of any glioma in patients with a history of diabetes. The inverse association was stronger among patients with type I diabetes (OR 0.59, 95% CI 0.24‐1.46) than among patients with not specified diabetes (OR 0.86, 95% CI 0.73‐1.00), but the results for type I diabetes were only based on five cases.

We also observed an inverse association between diabetes in general (type I and type II) and the risk of glioblastoma. Furthermore, longer duration of diabetes (type I and type II) was inversely related to the risk of glioma. Interestingly, a reduced risk of glioma was described previously in patients with diabetes both in case‐control studies 15, 30, 66, 67 and prospective cohort studies,^16^, ^68^, ^69^, ^70^ indicating that a high blood glucose level might be inversely related to glioma risk. Possible underlying mechanism have already been discussed in detail in these previous studies,^30^, ^69^ however, those studies often had no information on the type of diabetes.^15^, ^16^, ^30^, ^66^, ^67^, ^68^, ^70^

Several limitations of our study should be mentioned. We had no information on molecular alterations in patients with glioma, which are more accurate in describing the biological behavior of glioma subtypes than histology alone.^32^, ^71^ As these alterations have only been determined in recent years, they are not available in epidemiologic studies covering longer observation periods. Because the data are based on primary health care data and because general practitioners record the diagnoses, misclassification is possible. However, the accuracy of the CPRD GOLD has been repeatedly demonstrated.^27^ Adjustment for socioeconomic status and lifestyle factors was not possible because this kind of information is not regularly recorded in our database. Nonetheless, cases and controls were matched on general practitioner, which serves as a proxy for socioeconomic status since people from the same area are likely to be of comparable social class. Furthermore, other studies that assessed socioeconomic status did not find significant associations with glioma risk.^72^, ^73^ However, we have to point out that controls were more likely than cases to be smokers and have a low BMI. Smoking is known to be prevalent more often in lower socioeconomic classes.^74^ This could also explain why heart failure and myocardial infarction occurred more frequently in controls. It is therefore conceivable, that these patients exhibit higher levels of immune dysfunction which could affect prevalence of other immune‐related disorders in some way. We implemented smoking status and BMI as potential confounders into our multivariate model. Nonetheless, we cannot fully exclude confounding by socioeconomic status in our study. Finally, our results for lower grade glioma and younger patients with glioma were based on few cases only and must therefore be interpreted with caution.

Our study has several strengths. The CPRD contains medical records of more than 11 million people and has been found to be representative of the British population.^26^ In this large case‐control study, we included 3112 patients with glioma, more than most other studies investigating the effect of immune‐associated disorders on the incidence of gliomas. Unlike many other case‐control studies, the information analyzed in our study was not self‐reported, but collected prospectively by health care professionals in the absence of any study hypothesis, rendering the data more accurate and reducing potential recall‐bias. Nonetheless, it is possible that not all diagnoses such as for example, hay fever are properly reported to physicians, especially if patients have more serious diseases to discuss.

In conclusion, we found no material associations between autoimmune diseases, allergies, or immunosuppressive therapies and the risk of glioma. However, the risk of glioma was reduced in patients with longer duration of diabetes. Subgroup analyses in patients less than 40 years of age indicated a positive association between inflammatory bowel diseases and the risk of glioma. There was also a suggestion of an inverse relation between asthma and incidence of glioma especially in younger patients.

## Data Availability

Study data were obtained from CPRD primary care data under license from the UK Medicines and Healthcare products Regulatory Agency. All data are provided by patients and collected by the NHS as part of their care and support. The interpretation and conclusions contained in this study are those of the authors alone.
